# Vat Photopolymerization-Based Additive Manufacturing of Si_3_N_4_ Ceramic Structures: Printing Optimization, Debinding/Sintering, and Applications

**DOI:** 10.3390/ma18071556

**Published:** 2025-03-29

**Authors:** Zi-Heng Wang, Yun-Zhuo Zhang, Wei-Jian Miao, Fan-Bin Wu, Shu-Qi Wang, Jia-Hu Ouyang, Ya-Ming Wang, Yong-Chun Zou

**Affiliations:** School of Materials Science and Engineering, Harbin Institute of Technology, Harbin 150001, Chinayunzhuo_hit@163.com (Y.-Z.Z.); 23s009061@stu.hit.edu.cn (W.-J.M.); 23s109209@stu.hit.edu.cn (F.-B.W.); wangyaming@hit.edu.cn (Y.-M.W.); zouyongchun@hit.edu.cn (Y.-C.Z.)

**Keywords:** vat photopolymerization, Si_3_N_4_, debinding, sintering, shape/property precise regulation

## Abstract

Si_3_N_4_ ceramics and composites stand out for their exceptional mechanical and thermal properties. Compared with conventional ceramic forming processes, 3D printing via vat photopolymerization not only ensures high geometric precision but also improves the forming efficiency and strength of green body. Nevertheless, the grayish appearance of Si_3_N_4_ and its relatively high refractive index can adversely affect the photocuring behavior in ceramic slurries. The primary objectives focus on enhancing the curing performance and rheological properties of slurries, minimizing defects during post-processing, and improving the relative density and mechanical properties of Si_3_N_4_ ceramics. Key advancements include slurry optimization via refractive index matching, biomodal particle gradation and surface modification, while the integration of whisker/fiber additions or polymer-derived ceramic strategies enhances mechanical properties. In addition, controlling the atmosphere and heating rate of the post-processing innovations can achieve a relative density of more than 95%. This paper introduces the mechanisms of vat photopolymerization and then summarizes the strategies for improving Si_3_N_4_ ceramic slurries as well as controlling the printing and debinding/sintering processes. It further highlights the ways in which different approaches can be used to enhance the properties of Si_3_N_4_ slurries and ceramic parts. Finally, applications of Si_3_N_4_ ceramics and composites via vat photopolymerization in various fields such as aviation, aerospace, energy, electronics, chemical processes, and biomedical implants are also presented to point out future opportunities and challenges.

## 1. Introduction

Silicon nitride (Si_3_N_4_) stands out for its exceptional mechanical properties, including its high flexural strength, fracture toughness, hardness, wear resistance, thermal shock stability, low dielectric constant, and wave-transparency, making it indispensable in extreme environments [1,2]. Its applications span critical fields such as biomedical implants, aerospace components, and defense systems [3,4,5]. However, conventional ceramic fabrication methods, such as cold pressing, gel casting, and tape casting, face limitations in producing geometrically intricate Si_3_N_4_ parts due to mold-dependent processes, high costs, and challenges in achieving inter-connected porous or thin-walled structures [6,7].

Additive manufacturing (AM) has emerged as a transformative solution, enabling the mold-free production of complex geometries with high precision and efficiency [8,9]. By converting computer-aided designs into layer-by-layer fabrication paths, AM overcomes the bottlenecks of traditional machining and mold-based processes, accelerating design, prototyping, and optimization cycles [10]. AM has been widely used in various sectors like healthcare [11,12,13], cultural heritage preservation [14,15,16], and aerospace [17]. Over the decades, diverse AM techniques have been developed, including selective laser sintering/melting (SLS/M) [18,19], 3D printing (3DP) [20,21], stereolithography (SL) [22], direct ink writing (DIW) [23,24,25], and fused deposition modeling (FDM) [26,27,28]. These methods utilize thermal, laser, or ink-based mechanisms to shape powders, filaments, or slurries, offering advantages in customization, efficiency, and material conservation [29,30,31].

Among AM technologies, the vat photopolymerization-based ceramic process has gained prominence due to its high resolution, cost-effectiveness, and scalability, and it opens new avenues for complex-shaped ceramic parts [32,33]. While oxide ceramics like ZrO_2_ [34,35,36], Al_2_O_3_ [37,38], and SiO_2_ [39,40] have seen mature applications, research on gray/dark-colored ceramics such as Si_3_N_4_ [41,42], AlN [43], and SiC [44,45,46] or composites remains very limited. Challenges arise from the high refractive index (2.1) of Si_3_N_4_, which creates a mismatch with those (1.4–1.6) of UV-curable resins, reducing curing depth and resolution [47]. Additionally, its gray hue intensifies UV absorption, further impairing slurry photopolymerization [48]. Due to strong covalent bonding and low self-diffusivity, Si_3_N_4_ requires sintering aids for densification. This highlights the importance of optimizing slurry formulations and thermal processing.

Although some studies have explored the fabrication of Si_3_N_4_ ceramics via vat photopolymerization, there still remains a lack of comprehensive reviews that address both the improvements of Si_3_N_4_ slurry formulations and the optimization of printing, debinding/sintering protocols, and the methodology of shape/property precise regulation. This review examines the mechanisms of photopolymerization-based AM, strategies for enhancing Si_3_N_4_ slurry performance, including particle gradation, resin/dispersant selection, surface modification, multiphase ceramics, polymer-derived ceramics, and the impact of debinding/sintering processes. Applications and future challenges of vat photopolymerization-fabricated Si_3_N_4_ components are also discussed. This paper aims to provide guidance for advancing vat photopolymerization techniques in Si_3_N_4_ ceramics and composites, offering actionable strategies to address challenges and accelerate process innovation.

## 2. Principles of Ceramic 3D Printing via Vat Photopolymerization

Vat photopolymerization, one of the earliest and most refined 3D printing technologies, relies on the UV-induced polymerization of photosensitive resins. Light patterns projected onto the resin surface trigger free-radical decomposition, forming solidified layers with high precision and smooth surfaces [49]. Over the past 30 years, many efforts have been made to develop various techniques like stereolithography (SLA), digital light processing (DLP), and two-photon polymerization (TPP).

### 2.1. Stereolithography (SLA)

Stereolithography (SLA), pioneered by Hull et al. in 1986 and later commercialized by 3D Systems, revolutionized additive manufacturing by enabling high-precision layer-by-layer fabrication. This technique gained broader recognition in ceramic processing after Griffith and Halloran [50] extended their applications to ceramics, marking a milestone in photopolymerization-based ceramic shaping.

In the SLA process, the build platform first descends to a predefined layer thickness (typically 12–150 μm, with 100 μm being most common in practice). A recoating blade then spreads a uniform layer of photosensitive ceramic slurry across the platform. A UV laser beam selectively cures the resin according to sliced STL file templates, initiating photopolymerization in targeted regions. Following each layer’s curing, the platform adjusts vertically (either upward or downward depending on the printer configuration) to accommodate the next layer. This iterative layering continues until the complete 3D structure is formed [51].

**Figure 1:**
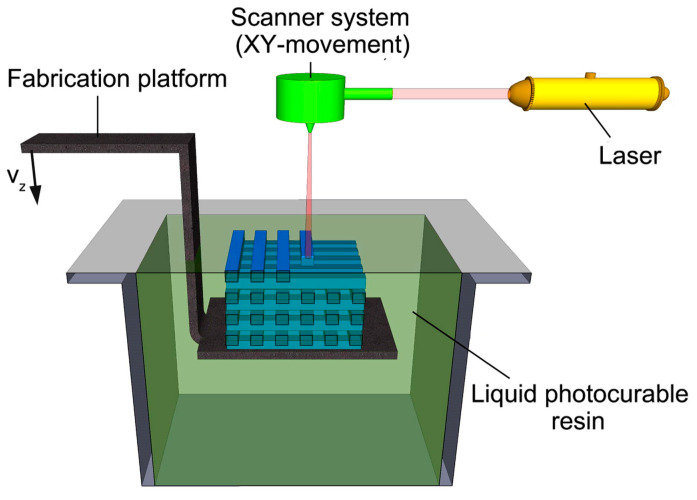
Schematic of stereolithography, adapted from reference [54], copyright 2012, with permission from Elsevier.

Standard SLA systems operate at speeds of 10–20 mm/hour, with resolution directly governed by the laser spot diameter at the curing point—typically tens of micrometers. The resin needs to be photoactive and transparent. While SLA achieves exceptional dimensional accuracy, its reliance on laser systems results in higher equipment costs and slower production rates compared to other additive techniques [52,53]. The schematic of stereolithography is shown in Figure 1 [54].

### 2.2. Digital Light Processing (DLP)

Digital Light Processing (DLP), first developed by Takagi et al. in 1993, shares fundamental principles with stereolithography (SLA) but employs a full-layer exposure strategy to cure entire resin layers simultaneously. This approach significantly reduces energy consumption and improves printing efficiency, earning it recognition as mask-based stereolithography or projection micro-stereolithography [55,56].

**Figure 2:**
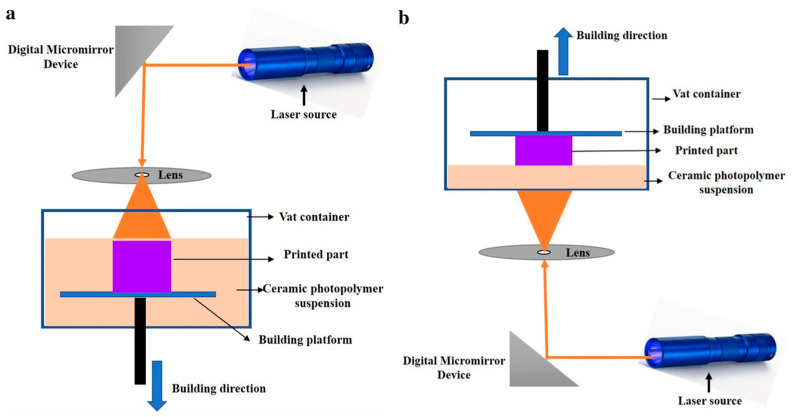
Schematic illustration of DLP 3D printing: (**a**) top-down apparatus; (**b**) bottom-up apparatus, adapted from reference [58], copyright 2022, with permission from Springer Nature.

At the core of DLP systems lies the digital micromirror device (DMD), developed by Texas Instruments (Texas, USA), which utilizes arrays of nearly one million micromirrors to project precise light patterns onto the resin surface and enables continuous high-resolution printing [57]. The process operates in two primary configurations, top-down and bottom-up approaches, as shown in Figure 2 [58].

Components produced via DLP exhibit smooth surfaces and high dimensional accuracy, with sintered parts demonstrating superior mechanical properties. The advantages of DLP, including low production costs, rapid prototyping, and resolutions as fine as 10 μm, have driven its widespread adoption in additive manufacturing [59]. A key benefit is its ability to fabricate ceramic parts without molds, offering unparalleled flexibility for manufacturing complex ceramic composites. This contributes to the position of DLP as a highly reliable, efficient, and economically viable method for the production of advanced ceramic components.

### 2.3. Two-Photon Polymerization (TPP)

**Figure 3:**
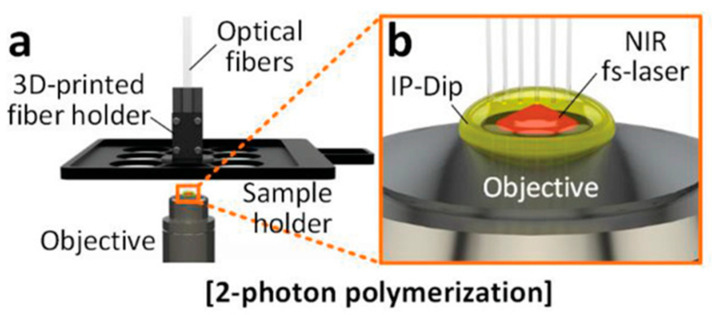
Schematic illustration of TPP: (**a**) system arrangement of the TPP objective, sample holder, and optical fibers clamped by a 3D-printed fiber holder; (**b**) detailed view of the configuration of the TPP objective, photoresist (IP-Dip), and optical fiber tips during the TPP process, adapted from reference [61], copyright 2020, with permission from John Wiley and Sons.

Two-photon polymerization (TPP) represents an advanced additive manufacturing technique that is capable of fabricating three-dimensional micro/nanostructures with sub-wavelength resolution. This process leverages the nonlinear optical phenomenon where a molecule simultaneously absorbs two photons to initiate photopolymerization, with the reaction predominantly confined to the focal volume of a femtosecond laser due to its quadratic intensity dependence [60]. TPP distinguishes itself from conventional layer-by-layer 3D printing methods by enabling the direct solidification of photocurable polymers at arbitrary depths within liquid resin, as shown in Figure 3 [61]. Through precise spatial control of the laser focus along preprogrammed CAD trajectories, this technique achieves dimensional accuracy surpassing conventional optical diffraction limits while eliminating the material deposition constraints that are inherent to traditional additive manufacturing approaches [62].

Despite these advantages, practical implementation of TPP still faces several technical constraints. TPP technology imposes optical transparency requirements on ceramic slurries, rendering the opaque ceramic suspensions commonly used in DLP systems incompatible with TPP fabrication. Furthermore, the extraordinary spatial resolution (typically <200 nm) necessitates extended fabrication durations and restricts practical applications to microscale components, presenting notable challenges in production efficiency for macroscopic structures.

### 2.4. Photopolymerization Mechanisms

**Figure 4:**
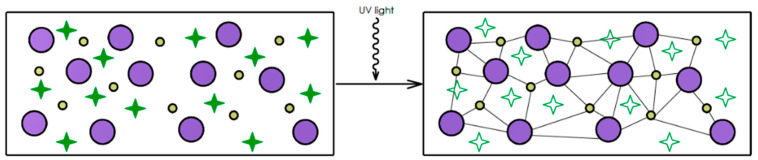
Liquid photopolymer (on the left) and polymerization induced by light (small circle—monomer, large circle—oligomer, star—photoinitiator), reprinted from reference [57], copyright 2021, with permission from MDPI.

The photopolymerization utilizes photosensitive resins which undergo a curing process when exposed to visible or ultraviolet (UV) radiation. Upon illumination, photoinitiators within the resin trigger polymerization reactions that form cross-linked polymer chains, ultimately solidifying the material. The photopolymer composite comprises three essential components: monomers, oligomers, and photoinitiators, as shown in Figure 4 [57]. In ceramic vat photopolymerization systems, UV or violet light sources induce electronic transitions between valence and conduction bands through photon absorption by the material [63]. When activated by the curing light, photoinitiators generate reactive species that catalyze chain propagation between monomers and oligomers. This thermally activated chain formation process progresses irreversibly. Finally, the liquid resin is permanently transformed into solid structures through layer-by-layer fabrication guided by sliced STL files [64].

**Figure 5:**
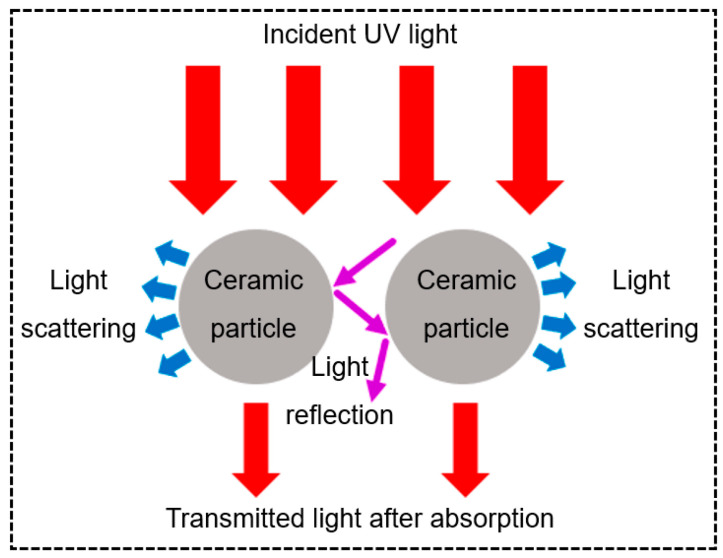
Light–particle interactions during the ceramic VP process, reprinted from reference [65], copyright 2024, with permission from MDPI.

Figure 5 shows four primary light–particle interactions during ceramic VPP processing: absorption, scattering, reflection, and transmission [65]. These phenomena collectively determine the penetration depth and curing characteristics of UV light. Increased ceramic particle loading enhances light absorption while reducing the effective penetration depth, creating competition between ceramic particles and photoinitiators for photon energy. Significant scattering effects not only cause energy dissipation but also induce edge blurring in cured regions, ultimately compromising printing resolution. However, surface reflections from ceramic particles combined with a reduced obstruction to light propagation can paradoxically improve the UV penetration depth under specific conditions, increasing the achievable curing thickness [65].

When fabricating ceramic components via the DLP method, the curing depth and over-curing width are critical parameters that significantly influence the final part accuracy. The Jacobs equation is commonly employed to determine the curing thickness of the VP slurry, according to the following equation [65]:

(1) $$D_{c}=D_{p}\cdot \ln\left(\frac{E_{0}}{E_{\mathrm{crit}}}\right)$$

where *D_c_* is the curing thickness, *D_p_* is the depth of light penetration in the ceramic VP slurry, *E*_0_ is the incident light intensity, and *E_crit_* is the critical light intensity. The difference in the refractive index between the ceramic powder and the monomer of the photosensitive resin determines the intensity of the scattering effect. The greater the difference between these two values, the stronger the scattering effect. Griffith et al. [66] proposed a relationship formula for calculating the transmission depth of a laser in the ceramic slurry, according to the following formula:

(2) $$D_{p}\text{∝}\frac{2d_{50}}{3}\frac{\lambda }{\phi }\frac{n_{0}^{2}}{{\text{∆}n}^{2}}$$

where *d*_50_ is the average particle size of the ceramic particles, λ is the wavelength of incident light, ϕ is the solid loading, *n*_0_ is the refractive index of the photosensitive resin, and ∆n is the difference between the refractive indices of the ceramic particles and the photosensitive resin. In Griffith’s theory, the curing depth is influenced by the ceramic solid phase content, particle size, and the refractive index difference between the ceramic powder and the photosensitive resin. Among these factors, the refractive index difference between the ceramic and the resin is the primary determinant.

However, for ceramic slurries, the curing width exceeds the laser beam diameter due to lateral scattering effects, thus requiring a correction coefficient to modify the conventional formula. In this case, Gentry et al. [67] divided the curing width (*C_w_*) into two components: the laser beam irradiation width (*W_beam_*) and the scattering-induced expansion width (*W_ex_*). The expansion width is expressed by the following formula:

(3) $$W_{\mathrm{ex}}=D_{w}\cdot \ln\left(\frac{E_{0}}{E_{w}}\right)$$

where *E_w_* is the critical exposure dose in the width direction, *D_w_* is the sensitivity coefficient of the resin to light in the width direction, and *E*_0_ is the incident light intensity. Since curing in the width direction is purely induced by scattering, the curing parameters *D_w_* and *E_w_* differ from those in the thickness direction (*D_p_* and *E_c_*). The sensitivity coefficient of the resin to light in the width direction is significantly larger [68]. The cross-sectional profile of the cured resin exhibits a parabolic shape. Based on geometric relationships, Jacobs derived an equation linking the curing width (*C_w_*) and curing thickness (*C_d_*), according to the following formula:

(4) $$C_{w}=W_{0}\sqrt{\frac{2C_{d}}{D_{p}}}$$

where *W*_0_ is the Gaussian radius of the laser spot. During the 3D printing process, when *W_0_* and *C_d_* remain constant, the curing width (*C_w_*) depends solely on the sensitivity coefficient *D_p_* in the thickness direction. Due to scattering effects caused by ceramic particles, the transmission depth of the ceramic slurry is smaller than that of conventional resin, which results in a larger curing width for the ceramic slurry compared to ordinary resin.

As the incident light intensity (*E*_0_) increases, the curing thickness initially grows proportionally. Beyond a critical threshold, this growth rate markedly declines and eventually plateaus, with further energy increments providing negligible improvements. Excessive light energy induces resin carbonization, which causes failure in the VPP process. In addition, excessive incident light intensity exacerbates the expansion width (*W_ex_*), which directly reduces the final part accuracy and elevates defect formation risks during post-processing, ultimately degrading both the mechanical properties and relative density of Si_3_N_4_ ceramics. Therefore, it is crucial to select the appropriate incident light intensity and exposure time based on the curing thickness and expansion width of the slurry to ensure the forming accuracy.

## 3. Strategies for Improving Si_3_N_4_ Ceramic Slurries

The photopolymerized ceramic green bodies require subsequent thermal treatments of debinding and sintering to achieve sufficient densification and mechanical strength, necessitating photopolymerizable suspensions with a high solid loading of higher than 50 vol.% [69]. To ensure successful layer-by-layer fabrication, these suspensions must maintain a viscosity below 3000 mPa·s with pseudoplastic behavior, demonstrating negligible yield stress to facilitate rapid recoating between layers.

**Figure 6:**
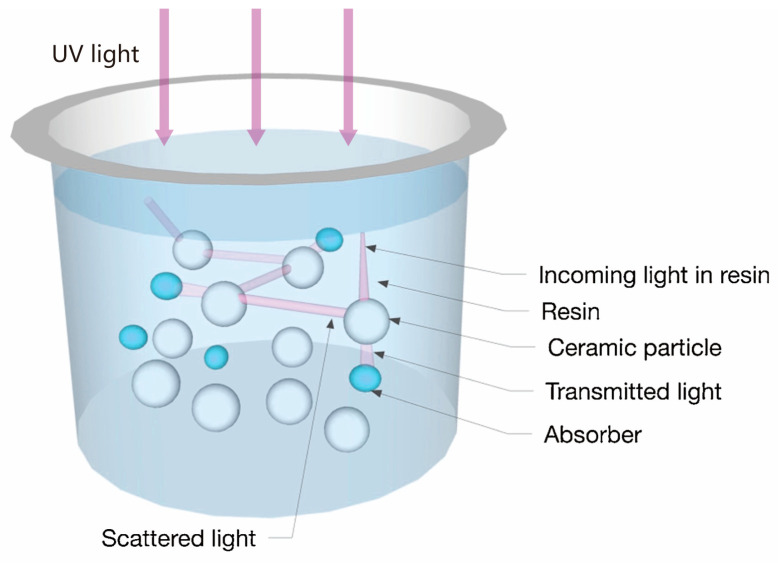
Simple view of the scattered light (only in one direction) and transmitted light as a light ray hits a suspended ceramic particle in liquid resin, adapted from reference [52], copyright 2020, with permission from Elsevier.

Effective formulation demands homogeneous ceramic particle distribution within the photosensitive resin matrix while maintaining appropriate rheological properties. UV-induced polymerization requires precise wavelength matching between the light source and the photoinitiator absorption spectrum, as shown in Figure 6 [52]. The suspension must exhibit sufficient optical transparency to permit UV penetration within defined parameters, enabling controlled polymerization at targeted depths.

Significant light attenuation occurs due to the refractive index mismatch between ceramic particles (*n* ~ 1.7–2.4) and organic resins (*n* ~ 1.5), particularly in high-solid-loading suspensions. This mismatch causes pronounced scattering effects that both reduce the curing depth and create energy competition between ceramic particles and photoinitiators [70]. While an increased ceramic content enhances the density of final parts, it paradoxically diminishes UV penetration, which typically limits the practical curing depth to <200 μm even with optimized formulations [71].

### 3.1. Particle Size Optimization

The particle size of silicon nitride ceramic powders significantly influences both the curing behavior and rheological properties of ceramic slurries. Many efforts have been made by focusing on ceramic powders with monomodal size distributions, where finer particles typically enhance the mechanical performance after sintering. Liu et al. [72] investigated the UV absorption characteristics of Si_3_N_4_ particles with varying colors and sizes, and found that the darker-colored slurries exhibited a poorer light transmission and curing efficiency. For particles of identical size, lighter-colored Si_3_N_4_ slurries demonstrated a lower scattering coefficient, for example, gray powders with an average size of 0.8 μm achieve a minimal scattering coefficient of 202. Larger white particles with an average size of 2.0 μm further reduce the scattering coefficient to 110. Si_3_N_4_ ceramic slurries prepared with a particle size of 800 nm achieve an optimal stability and a curing depth of 0.045 mm.

However, a monomodal distribution causes inherent limitations: fine particles increase the viscosity of the ceramic slurry and reduce the curing depth at equivalent solid loading. This has driven the exploration of particle gradation strategies to optimize the fabrication of Si_3_N_4_ ceramics. Key advantages of a bimodal size distribution include reduced viscosity for higher solid loading, an enhanced curing depth to improve the printing efficiency, and microstructural control for superior mechanical strength. Huang et al. [73] studied the slurries with α-Si₃N_4_ powders in two different particle sizes of 1 μm and 2.7 μm, and found that increasing the content of fine particles led to an enhanced viscosity, a reduced curing depth, and an improved slurry stability through inter-particle filling effects.

Zhao et al. [74] demonstrated that a 7:3 ratio of fine (0.5 μm) to coarse (2.7 μm) bimodal particle gradation enables 45 vol.% ceramic slurries with a viscosity of 5.4 Pa·s at a shear rate of 30 s^−1^, which results in a cure depth of 42 μm under the exposure of 288 mJ/cm^2^. The resulting sintered components exhibited a flexural strength of 679 MPa, representing a 26.92% improvement over monomodal systems. This approach was successfully used to fabricate Si_3_N_4_ impellers via DLP for high-temperature hot-section applications. Notably, while bimodal distributions reduce the viscosity, they concurrently decrease the curing thickness, necessitating balanced formulation design. Li et al. [47] investigated the influence of particle size distribution by introducing 5 μm Si particles to 0.5 μm Si_3_N_4_ slurries, illustrating the tradeoff between viscosity reduction and a diminished curing capacity in hybrid systems.

Traditional forming techniques like dry pressing and cold isostatic pressing have explored the modification of Si_3_N_4_ ceramics through the introduction of *β*-Si_3_N_4_ seed crystals with a volume fraction of 5–10 vol.% into *α*-phase dominant powders [75,76]. These seeds act as nucleation sites, promoting the growth of elongated *β*-Si_3_N_4_ grains that create bimodal microstructures and significantly improve mechanical performance. During sintering, dissolved *α*-Si_3_N_4_ preferentially precipitates both as fine *β*-grains and epitaxial layers on existing *β*-seeds, facilitating controlled microstructural evolution.

**Figure 7:**
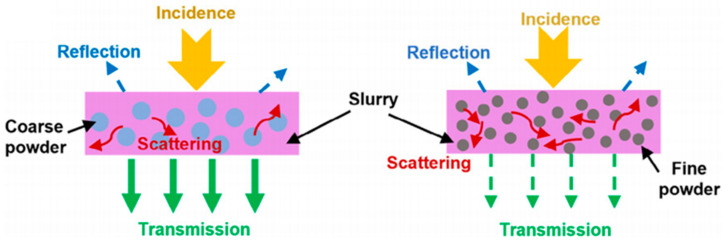
Schematic illustration of the interaction between particles and light, adapted from reference [77], copyright 2024, with permission from John Wiley and Sons.

Wang et al. [77] optimized this approach by using a bimodal particle blend of 0.5 μm *α*-Si_3_N_4_ and 3 μm *β*-Si_3_N_4_ powders at a 50 vol.% solid loading. The *α*:*β* Si_3_N_4_ mass ratio of 2:8 achieves a minimal viscosity of 23 Pa·s at a shear rate of 30 s^−1^ and an improved suspension stability through enhanced particle packing, although increasing the *α*-content reduced the curing depth by 18%. A *α*:*β* Si_3_N_4_ mass ratio of 4:6 balanced these properties, which resulted in sintered Si_3_N_4_ ceramics with a flexural strength of 470 MPa through controlled grain growth. Figure 7 shows the schematic illustration of the interaction between particles and light [77].

Mao et al. [78] innovatively combined vat photopolymerization (VPP) with seeding strategies, and revealed a nonlinear viscosity response to the *β*-seed content of 15–35 vol.%. Initial viscosity increases (up to 28% at a 20 vol.% *β*-seed content) stemmed from particle entanglement by elongated *β*-grains, while higher *β*-seed concentrations of more than 25 vol.% reduce the specific surface area, decrease the inter-particle friction, and ultimately lower the viscosity by 12–15%. This approach is able to realize precise microstructural control, which demonstrates the viability of photopolymerization-based additive manufacturing for engineering-grade Si_3_N_4_ components.

### 3.2. Resin and Dispersant Selection

The inherent challenges of silicon nitride in stereolithography are its high refractive index and UV absorption, which significantly constrain the curing depth. To mitigate light scattering, researchers have developed high-refractive-index (RI) resin systems that reduce the RI mismatch between ceramic particles and organic matrices. The primary purpose of dispersants is to inhibit the particle agglomeration of ceramic powders through electrostatic or steric repulsion stabilization mechanisms [79]. Mechanistically, the acidic functional groups in dispersants, such as BYK-110 and Solsperse 41000, serve as molecular anchors through chemisorption onto Si_3_N_4_ particle surfaces. Concurrently, the hydrophilic chains enhance particle wettability while establishing spatial barriers within the resin matrix, thereby promoting powder dispersion through combined electrostatic and steric stabilization effects. Nevertheless, excess dispersant can increase the viscosity of the slurry. This phenomenon arises from intermolecular crosslinking among surplus dispersant molecules, which induces unintended polymer entanglement and particle agglomeration. Table 1 summarizes the viscosity and curing depth of different Si_3_N_4_ slurry formulations. Chung et al. [80] pioneered this approach by selecting 1,6-hexanediol diacrylate (HDDA) as the optimal monomer for low-viscosity Si_3_N_4_ slurries, leveraging its balanced polarity and hydrogen-bonding parameters. Li et al. [47] also demonstrated the feasibility of HDDA-based slurries with a solid loading of 35 vol.% to achieve stable printing performance.

The trifunctional TMPTA creates multiple crosslinking sites that accelerate the development of compact polymer networks. However, excessive TMPTA concentrations may lead to strong interfacial adhesion between cured components and the substrate, potentially causing demolding challenges during post-processing. In contrast, the monofunctional HEMA and difunctional HDDA introduce flexible molecular segments that effectively modulate crosslinking density. Nevertheless, excessive HEMA and HDDA concentrations can also increase the elongation at break of the cured films. Shen et al. [81] investigated the influence of a mixture of HEMA, HDDA, and TMPTA in a volume ratio of 3:4:3 with a Si_3_N_4_ loading of 48 vol.%, and achieved a viscosity of 2.09 Pa·s at a shear rate of 30 s⁻^1^. This slurry exhibited a curing depth of 80 μm at an exposure energy of 126.09 mJ/cm^2^ with minimal sedimentation of less than 2% over 24 h.

Lin et al. [82] investigated the influence of monomer functionality using HDDA, trimethylolpropane triacrylate (TMPTA), and pentaerythritol triacrylate (PPTTA). Their findings revealed that higher-functionality monomers enhance stability and curing depth (up to 28% improvement with TMPTA) at the cost of increased viscosity (2.3-fold higher than HDDA) and over-curing width. The tetrafunctional PPTTA improves slurry curing efficiency, although its higher molecular weight elevates the viscosity of the slurry. Dispersant optimization using 1–2 wt.% BYK110 has proved to be critical for maintaining homogeneity in these systems.

**Table 1:** Summary of viscosities and curing depths of different Si_3_N_4_ slurry formulations.

Resin	D_50 _ (μm)	Dispersant	Solid Loading (vol.%)	Viscosity (Pa·s)	Curing Depth (μm)	Ref.
HDDA	0.5	BYK-103 (3 wt.%)	42	2.8 at 100 s^−1^	38	[47]
HEMA:HDDA:TMPTA = 3:4:3 (vol.%)	0.5	BYK-110 (1 wt.%)	48	2.09 at 30 s^−1^	80	[81]
HDDA:ACMO:POE = 5:2:3 (wt.%)	0.45	KD1 (2.5 wt.%) + CC42 (0.5 wt.%)	40	1.9 at 6.4 s^−1^	38	[83]
OPPEA:ACMO:HDDA:POE = 2:1:4:3 (wt.%)	0.45	KD1 (2.5 wt.%) + CC42 (0.5 wt.%)	40	4.3 at 6.4 s^−1^	<45	[84]
PPTTA:OPPEA = 4:3 (wt.%)	0.813	Solsperse 85000 (3 wt.%)	20	4.19 at 1.18 s^−1^	60.75	[85]
HDDA + TMP3EOTA	0.83	Solsperse 41000	40	<1.5 at 60 s^−1^	61.6	[86]

For LCD-based masking systems, Wu et al. [83] developed a 40 vol.% Si_3_N_4_ slurry using ACMO/POE additives, which reduced required exposure energy by 45% and maintained a cure depth of 38 μm at an exposure energy of 56.8 mJ/cm^2^. Among monofunctional acrylates, ACMO offers rapid curing, low odor, and stable volatility. Its refractive index surpasses that of HDDA, while HEMA enhances adhesion via hydroxyl groups. POE stands out with a benzene ring structure that boosts the refractive index and improves KD1 dispersant compatibility. The optimized HDDA: ACMO: POE blend in a weight ratio of 5:2:3 exhibited a shear-thinning behavior with a viscosity below 1900 mPa·s above a shear rate of 6.4 s^−1^, demonstrating an enhanced printability for complex geometries.

Wu et al. [84] pioneered a refractive index-matched photocurable slurry by developing a high-RI liquid phase (*n* ≈ 1.68) to minimize optical scattering at interfaces between Si_3_N_4_ and resin. Their optimized formulation combined 40 wt% HDDA, 20 wt% OPPEA (a high-RI acrylate), 10 wt% ACMO, and 30 wt% POE (non-reactive high-RI solvent), enabling 40 vol.% Si_3_N_4_ slurries with enhanced curing depths and dimensional accuracy. Finite element modeling confirmed the improved UV intensity distribution within these index-matched systems, demonstrating a 32% greater light penetration as compared with conventional HDDA-based slurries.

Zou et al. [85] optimized both resin chemistry and dispersant selection for high-solid-loading Si_3_N_4_ slurries. OPPEOA (*n* ≈ 1.72) was identified as the most effective high-RI monomer, achieving exceptional curing sensitivity (*S_d_* = 16.52 μm) with remarkably low critical energy (*E_d_* = 8.50 mJ/cm^2^). At a Solsperse 85000 dispersant concentration of 3 wt.%, slurries with 44 vol.% Si_3_N_4_ loading exhibited optimal rheology with a viscosity of 4.19 Pa·s at a shear rate of 1.18 s^−1^, while maintaining stability over 72 h. This dual optimization of optical and flow properties enables the reliable fabrication of high-density Si_3_N_4_ components, bridging the gap between theoretical modeling and practical manufacturability in vat photopolymerization-based ceramics.

These advancements highlight the delicate balance required between refractive index matching, monomer chemistry, and particle stabilization to enable high-performance Si_3_N_4_ fabrication via photopolymerization. Current formulations now achieve commercial-grade solid loadings of higher than 45 vol.% without adversely affecting critical rheological and optical properties for precision additive manufacturing.

### 3.3. Surface Modification

Recent advancements in surface engineering have significantly improved the processing and performance of silicon nitride ceramics. Table 2 summarizes the viscosity and curing properties of Si_3_N_4_ slurries with different particle surface modifications. Surface oxidation of non-oxide ceramic powders has emerged as a key technique for optimizing photopolymerization behavior. Chen et al. [87] demonstrated that the thermal oxidation of Si_3_N_4_ powders at 1200 °C for 1 h introduces a silica-rich layer, enabling 50 vol.% solid-loaded slurries with enhanced light penetration. This approach achieves an unprecedented layer thickness of 50 μm in DLP printing, doubling production efficiency when compared with conventional methods. Huang et al. [88] quantified these benefits, and found that the improvements in the cured depths of Si_3_N_4_ slurries from 34 μm (raw powder) to 51 μm (partially oxidized powders) at an exposure energy of 500 mJ/cm^2^, directly correlate with the reduced refractive index mismatch.

The refractive index principle was verified by Li et al. [89], who observed a 40% reduction in UV absorption after the surface oxidized treatment of raw Si_3_N_4_ powders. While this enhances the curing depth by 28–35%, it simultaneously increases the over-curing width by 12–18%, revealing a critical tradeoff between penetration capability and dimensional accuracy. Their work demonstrated that the absorption characteristics dominate the resolution control in DLP processes more significantly than the refractive index difference.

Although surface oxidation treatment effectively improves the curing performance of ceramic slurries, excessive oxidation significantly degrades the mechanical properties of final ceramic components. The silane coupling agent can directly be used as the surface modifier and dispersant. It facilitates the formulation of Si_3_N_4_ ceramic slurries with high solid loading, low viscosity, and large curing depths. Liu et al. [93] demonstrated the effectiveness of silane coupling agents as dual-functional dispersants, where KH560 formed covalent bonds with ethyl acrylate (EA) to create a thin polymer shell on Si_3_N_4_ particles. This modification reduces the refractive index mismatch by two orders of magnitude, enabling slurries with high solid loading, low viscosity, and a 25% increase in curing depth as compared with untreated systems. Yang et al. [94] demonstrated a synergistic approach of combining thermal oxidation at 1200 °C for 1 h and silane coupling with 1 wt.% KH560 to optimize the properties of an Si_3_N_4_ slurry. The oxidation process introduced silica phases that improved light penetration, achieving a curing depth of 65 µm. KH560 facilitated covalent bonding between particles and resins, enabling slurries with a solid loading of 50 vol.% and a viscosity of 1.2 Pa·s at a shear rate of 30 s⁻^1^. Post-processing of these slurries yielded dense Si_3_N_4_ components via DLP with a dimensional deviation of less than 2%, showcasing the effectiveness of dual surface modification.

Lu et al. [90] introduced a novel block copolymer of KMT-3331 for surface modification, achieving stable hydrogen-bond adsorption on Si_3_N_4_ particles at a loading of 2 wt.%. Combined with the lubricating effect of low-molecular-weight BYK 110 dispersant, this approach facilitated the preparation of 60 vol.% slurries with excellent flow properties.

Sun et al. [95] leveraged tetramethylammonium hydroxide (TMAH) to enhance the colloidal stability in porous Si₃N₄ fabrication. The TMAH-modified slurries exhibited a zeta potential of −100 mV at a pH of 12, reducing viscosity by 10–20% and increasing the curing depth by 10 μm through improved particle dispersion and light penetration. Sha et al. [42] highlighted glycerol as an effective refractive index modifier (*n* = 1.474), optimizing curing depth while maintaining slurry stability at 0.5 wt.% dispersant loading. Their study on oxidation parameters further revealed that extended oxidation durations at 1200 °C linearly improved curing performance, with 3 h treatments achieving a penetration depth of 45 μm, which is 32% greater than non-oxidized controls.

These developments complement traditional surface treatments like laser shock peening and ion bombardment [96], collectively advancing the frontier of high-performance ceramic fabrication through tailored interface engineering. Current surface-modified Si_3_N_4_ slurries now achieve commercial-grade solid loadings of 45–50 vol.%, while maintaining a viscosity of less than 5 Pa·s for precision printing applications.

**Figure 8:**
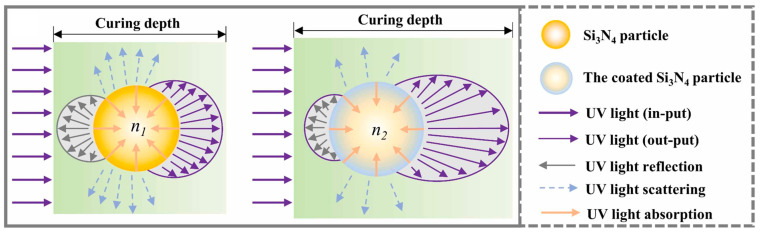
Schematic diagram of the light curing process of a Si_3_N_4_ ceramic slurry before and after coating, adapted from reference [91], copyright 2025, with permission from Elsevier.

Innovative coating technologies have also expanded beyond oxidation. Li et al. [91] developed a non-aqueous chemical precipitation (NCP) method for yttrium aluminum garnet (YAG) coatings on Si_3_N_4_ particles. The 200 nm-thick YAG layer reduces the slurry viscosity by 42.25% at a shear rate of 35 s^−1^, while boosting the cured depth to 35.4 μm (22.49% improvement) in a 30 vol.% Si_3_N_4_ ceramic slurry system. This dual enhancement of flow behavior and photoresponse underscores the potential of hybrid surface modification strategies in ceramic additive manufacturing. The absorbance and the refractive index of the Si_3_N_4_ powder can be reduced by the NCP process, which further leads to the reduction of the absorption, scattering, and reflection of UV light, as shown in Figure 8 [91]. The coated Si_3_N_4_ particle effectively reduces the difference between the refractive indices of the ceramic particles and the photosensitive resin, which can enhance the curing thickness of the slurry. In addition, the coated YAG can act as a sintering aid, which enhances the mechanical properties and relative density of the Si_3_N_4_ ceramics.

**Figure 9:**
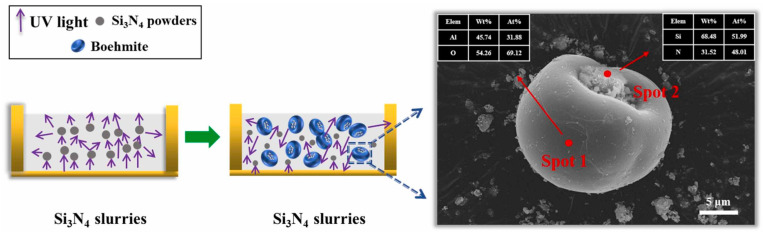
Schematic illustration showing the effect of a boehmite coating in the photocuring process, reprinted from reference [97], copyright 2024, with permission from Elsevier.

Wang et al. [92,97] developed multifunctional alumina coating strategies that simultaneously addressed processing and sintering challenges, as shown in Figure 9 [97]. Si_3_N_4_ powders are coated by bowl-like boehmite microspheres, which reduce the difference between the refractive indices of the ceramic particles and resin. This structure allows more UV light to penetrate the slurries and reduces the loss of exposure energy to improve the photocuring ability. The 2.5 wt.% boehmite-coated Si_3_N_4_ formulation achieves good mechanical properties, including a relative density of 93.15% and a flexural strength of 406.5 MPa, while enabling a precise control over porosity of 6.84–13.23% for tailored applications. This dual-purpose coating reduces light scattering at ceramic–resin interfaces and serves as an in situ sintering aid, which demonstrates the potential of integrated surface engineering for high-performance ceramic manufacturing.

Zhou et al. [86] developed a removable thermoplastic resin coating that reduced UV absorption by 22%, without adversely lowering the purity of sintered ceramics. At 10 wt.% coating content, slurries achieved a curing depth of 61.6 µm with excellent stability, while optimized 2 wt.% E51 resin formulations produced components with a flexural strength of 382.67 MPa and a density of 2.95 g/cm^3^ through vacuum-assisted sintering.

Li et al. [41] employed chemical co-precipitation to coat Si_3_N_4_ particles with Al_2_O_3_-Y_2_O_3_ sintering aids, which reduces the UV absorption by 18% and achieves a remarkably low viscosity of 0.042 Pa·s at a 30 s^−1^ shear rate with a precursor solution of 20 mL/100 g. The coated powders enabled a curing depth of 47.9 µm, representing a 35% improvement over unmodified systems, while maintaining a dimensional accuracy of 1.5% in complex architectures. The above-mentioned studies illustrate how advanced surface modification techniques can overcome historical limitations in vat photopolymerization-based ceramics, enabling the production of complex Si_3_N_4_ components with an enhanced resolution, mechanical strength, and process efficiency.

Further advancing surface engineering, Li et al. [98] leveraged AlN hydrolysis to create in situ Al_2_O_3_ coatings, addressing both optical (Δ*n* < 0.15) and processing challenges. The optimized slurry produced green bodies with a relative density of 92.6%, which were sintered into *β*-Si_3_N_4_/*β*-SiAlON composites with exceptional multifunctionality such as a flexural strength of 402.9 MPa, a hardness of 21.1 GPa, and a thermal conductivity of 37.4 W·m^−1^·K^−1^. This work established reactive surface modifications as a viable pathway for high-performance, thermally conductive ceramic components.

These innovations highlight the critical role of surface engineering in balancing optical, rheological, and mechanical requirements for silicon nitride additive manufacturing, enabling the production of complex, high-density components with tailored performance characteristics.

### 3.4. Multiphase Ceramics

The integration of ceramic matrix composites (CMCs) has emerged as a promising strategy to overcome mechanical limitations in additive-manufactured ceramics [99]. The addition of various whiskers or fibers can significantly enhance fracture toughness and strength. Furthermore, previous studies demonstrated that chemical vapor infiltration (CVI) can increase the density of preforms [100,101]. Mao et al. [102] demonstrated that zirconia (ZrO_2_) additions into Si_3_N_4_ systems enhance both processing and performance. At a ZrO_2_ content of 10 wt.%, flexural strength and fracture toughness increased by 61% from 214.7 MPa to 345.0 MPa and by 34% from 4.23 MPa·m^1/2^ to 5.69 MPa·m^1/2^, respectively, while simultaneously improving the curing depth from 25.2 μm to 64.8 μm through reduced UV absorption.

Sialon ceramics are solid-solution materials composed of silicon, aluminum, oxygen, and nitrogen in varying ratios. The substitution of Al-O bonds for Si-N bonds within their crystal structure endows sialon ceramics with superior thermal shock resistance and oxidation stability as compared with conventional silicon nitride ceramics. AlN-modified SiAlON ceramics via vat photopolymerization have a peak density of 3.17 g/cm^3^ and a thermal conductivity of 37.4 W·m^−1^·K^−1^ with the addition of 15 vol.% AlN, though the excessive addition of more than 20 vol.% AlN induces the formation of a detrimental *α*-phase [103]. Qin et al. [104] developed porous *β*-Si_3_N_4_/Si_5_AlON_7_ composites via DLP and liquid-phase sintering. With 30% SiO_2_ content, the material exhibited exceptional multifunctionality, such as a flexural strength of 149.2 MPa, a hardness of 11.8 GPa, and microwave-transparent dielectric properties such as a dielectric constant of 4.02 and a dielectric loss of 0.11 at 10 GHz, positioning it as a candidate for aerospace radome applications.

The mechanical properties of vat-photopolymerization-based Si_3_N_4_ ceramics can be enhanced through the strategic incorporation of nanoparticles and whiskers as reinforcing fillers. Vidakis et al. [105] enhanced biomedical resins through the incorporation of a 1.0 wt.% Si_3_N_4_ nanoparticle, achieving a 44.8% higher flexural strength and a 49.7% lower dimensional deviation than unmodified pure resin. Wang et al. [106] investigated the influence of carbon fiber incorporation on the mechanical properties of Si_3_N_4_ ceramics based on vat photopolymerization combined with precursor infiltration, pyrolysis, and found that carbon fiber-reinforced Si_3_N_4_ exhibited a 92.3% higher fracture toughness, which increases to 6.2 MPa·m^1/2^ at a C_f_ fiber loading of 6 wt.%, due to fiber bridging and interfacial pinning effects.

Functionally graded Al_2_O_3_-Si_3_N_4_ components via the stereolithography 3D printing reported by Xing et al. [107] showcased the correlation between viscosity and curing depth at 47 vol.%, with optimized dispersant levels (1.2–2%) enabling uniform 13,000 mPa·s viscosity and defect-free 40 μm layer fabrication.

Wang et al. [108] produced Si_3_N_4f_/Si_3_N_4_ composites with an enhanced toughness of 4.24 MPa·m^1/2^ and a radar-transparent dielectric constant (*ε* = 4) via the VPP-CVI approach. The hBN/*β*-Si_3_N_4_ hybrid filler system reported by Zhou et al. [109] enabled 3D-printed gyroid structures with a thermal conductivity of 1.42 W·m^−1^·K^−1^ at a solid loading of 60 wt.%, which demonstrated the potential for thermal management components.

These advancements illustrate how the compositing strategy with the reinforcements synergizes with additive manufacturing to produce Si_3_N_4_ ceramics with tailored mechanical, thermal, and functional properties. By optimizing reinforcement types (whiskers, fibers, nanoparticles), content ratios, and hybrid processing routes, researchers are overcoming traditional limitations in ceramic AM to expand application horizons.

### 3.5. Polymer-Derived Ceramics

Since their inception in the 1960s, polymer-derived ceramics (PDCs) have offered a unique pathway to ceramic fabrication through the pyrolysis of preceramic polymers like polysilazanes and polycarbosilanes [101]. This approach enables the direct shaping of complex geometries via 3D printing techniques such as SLA and DLP, bypassing the need for sintering aids while operating at lower temperatures of 700–1200 °C than conventional powder methods [110]. However, challenges persist in managing the substantial linear shrinkage of higher than 30% and the mass loss during pyrolysis, which often lead to structural defects [32,111].

Incorporating inert fillers such as particles, whiskers, or fibers has proven effective in mitigating shrinkage and improving mechanical performance [112,113]. Huang et al. [114] demonstrated this by dispersing Si_3_N_4_ particles and whiskers in a polysilazane matrix, achieving SiCN composites with a flexural strength of 180.7 MPa at 10 wt.% whisker loading. Excessive filler content of more than 15 wt.%, however, introduces porosity and reduces matrix continuity, which highlights the need for optimized reinforcement ratios. Li et al. [115] advanced this concept by developing DLP-printable slurries containing 60 wt.% Si_3_N_4_ whiskers in a polyborosilazane precursor. The resulting SiBCN/Si_3_N_4_ composites exhibited a remarkably low in-plane shrinkage of 18% during pyrolysis, retaining a dimensional fidelity in intricate architectures while reaching a flexural strength of 183 MPa after post-sintering at 1200 °C.

Wang et al. [116] combined DLP printing with precursor pyrolysis to fabricate both simple and honeycomb Si_3_N_4_ structures. Their photosensitive resin-polysilazane hybrids yielded ceramics with distinct mechanical profiles: 2D honeycombs showed a compressive strength of 65.5 MPa and an elastic modulus of 768.5 MPa.

These developments underscore the dual benefits of filler-reinforced PDCs with reduced processing defects and enhanced mechanical properties—while maintaining the inherent advantages of polymer-derived ceramic synthesis. Current research continues to refine the filler distribution, precursor chemistry, and pyrolysis protocols to expand the application scope of 3D-printed Si_3_N_4_-based ceramics in demanding thermal and structural environments.

## 4. Debinding/Sintering Process and Optimization for Si_3_N_4_ via VPP

### 4.1. Debinding Process

Although photopolymerization-based additive manufacturing enables complex Si_3_N_4_-based ceramic geometries, the technique faces inherent limitations in achievable wall thickness and post-processing requirements. The critical debinding step, such as the thermal removal of organic binders, must balance pyrolysis kinetics with structural integrity, as improper temperature profiles or atmospheres may induce cracking through gas pressure buildup [117,118].

Therefore, the design of thermal debinding processes is crucial. Currently, most researchers rely on thermogravimetric-differential scanning calorimetry (TG-DSC) to guide the design of the thermal debinding process, as it provides insights into the resin pyrolysis extent and endothermic/exothermic phenomena across temperature variations. Furthermore, derivative thermogravimetry (DTG) and second-derivative thermogravimetry (DDTG), derived from TG curves, can reveal the temperature of the maximum rate and the increase for binder removal. These characteristic points are instrumental in determining the holding temperature, holding time, and heating rate. For instance, prior to the temperature for exhibiting intense pyrolysis, reducing the heating rate and prolonging the holding time can stabilize gas generation and evacuation, thereby suppressing defect formation. However, even after establishing initial process parameters, iterative experimentation and parameter adjustments remain quite necessary. This empirical approach inevitably consumes significant resources and time, constituting a major challenge in current research.

The viable solution lies in developing a deeper mechanistic understanding of thermal debinding and establishing predictive models. Unfortunately, such studies remain quite scarce due to the inherent complexity of the process. Thermal debinding originates from the pyrolysis of cured resin, which requires appropriate kinetic models to describe the process and help define the mass conservation and energy conservation for subsequent modeling. There are many kinetic calculation methods for organic pyrolysis, including the Coats–Redfern method, the distributed activation energy model (DAEM), and the independent parallel reaction (IPR) method. The Coats–Redfern method simplifies pyrolysis into a single reaction process and obtains the average activation energy from non-isothermal thermogravimetric data [119]. Yan et al. [120] applied this method to study the kinetics process of thermal debinding in a novel paste injection 3D printing technique. The fitting result is satisfactory with the order of reaction assumed to be 1. However, the binder in their study is only a single-component paraffin. In contrast, pyrolysis is considered to contain multiple components or stages in DAEM and IPR methods. As a consequence, they potentially offer more superior fitting for complex resin networks composed of diverse monomers/prepolymers.

**Figure 10:**
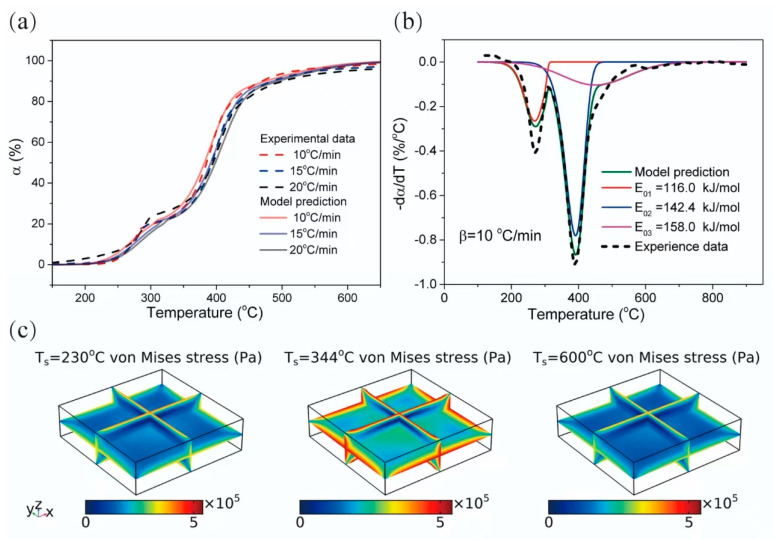
Model prediction of (**a**) the conversion rate α and (**b**) dα/dT compared with experimental data. (**c**) Model prediction of von Mises stress inside the gel-casting SiAlON green body at different temperatures, adapted from reference [122], copyright 2019, with permission from Elsevier.

In the DAEM method, the pyrolysis is regarded as the superposition of *n* independent pseudo-components, and the activation energy of each pseudo-component conforms to the normal distribution as follows [121]:

(5) $$\alpha =1-\int_{0}^{\text{∞}}\exp\left[-\frac{A}{\beta }\Psi \left(E,T\right)\right]f\left(E\right)dE$$

(6) $$\Psi \left(E,T\right)=\int_{T_{0}}^{T}\exp\left(-\frac{E}{\mathrm{RT}}\right)dT$$

where *α* is the conversion rate, *β* is the heating rate, *A* is the frequency factor, *E* is the activation energy, *R* is the gas constant, *f*(*E*) is the distribution of activation energy, and Ψ(*E*,*T*) is called the integral of the Boltzmann factor. For a system containing *n* pseudo-components, it satisfies the following equation:

(7) $$f(E)=\sum_{i=1}^{n}c_{i}f\left(E_{0i},\sigma_{i}\right),\;\sum_{i=1}^{n}c_{i}=1$$

where *c_i_* and *f*(*E*_0*i*_*,σ_i_*) are the proportion and distribution of activation energy of each pseudo-component, respectively. Li et al. [122] investigated the thermal debinding kinetics of the SiAlON green body prepared by gel-casting using the DAEM. The conversion *α* under different heating rates and reaction rates d*α*/d*T* predicted by the model aligned well with experimental results, as illustrated in Figure 10a,b [122]. Subsequently, the pyrolysis kinetic model was integrated into a solid/fluid thermal–mechanical coupling numerical model through finite element methods. As shown in Figure 10c [122], the von Mises stress peak in a green body was considered to be the primary cause of cracking during thermal debinding. It must be emphasized that optimizing the heating rate and holding process to suppress the maximum von Mises stress constitutes an important strategy for developing a suitable debinding process. Cui et al. [123] calculated the pyrolysis kinetic parameters for alumina green bodies prepared by stereolithography under an argon atmosphere using the DAEM as a comparison with three other methods, where the DAEM exhibited the best performance. The model demonstrates that the apparent activation energy increases with the conversion rate.

The IPR method divides the pyrolysis process into a weighted sum of several independent first- or n-th-order reactions. The overall kinetic parameters of the pyrolysis process can be represented as the weighted sum of parameters from individual sub-stages as follows:

(8) $$\frac{d\alpha_{i}}{dT}=\frac{k_{0,i}}{\beta }\exp(-\frac{E_{i}}{\mathrm{RT}})f_{i}\left(\alpha_{i}\right)$$

(9) $$w_{i}=\frac{\left(m_{i0}-m_{\mathrm{if}}\right)}{\left(m_{0}-m_{f}\right)},\sum_{i=1}^{N}w_{i}=1$$

(10) $$\alpha =\frac{\left(m_{0}-m_{T}\right)}{\left(m_{0}-m_{f}\right)}=\sum_{i=1}^{N}w_{i}\alpha_{i}$$

(11) $$E=\sum_{i=1}^{N}w_{i}E_{i}$$

where α*_i_*, *k*_0*,i*_, *E_i_*, *f_i_*(*α_i_*), *w_i_*, *m_i_*_0_, and *m_if_* are the conversion rate, pre-exponential factor, activation energy, reaction mechanism function, weighting factor, initial weight, and final weight for stage *i*, respectively. *α*, *m*_0_, *m_f_*, *m_T_*, and *E* represent the conversion rate, initial weight, final weight, mass of the gel polymer at temperature *T*, and apparent activation energy for the overall pyrolysis reaction process. *N* is the total number of sub-stages.

The pyrolysis of DMAA/MBAM polymers in gel-casting ceramic components was studied by Li et al. [124]. An activation energy variable model and several model-free methods were employed to calculate kinetic parameters for individual sub-stages within the IPR method, with the Šesták–Berggren (SB) model used to determine the reaction mechanism function. Further comparative studies indicated that the IPR method integrated with the SB model had better performance than the DAEM. It was asserted that this refined IPR method can achieve better fitting accuracy for the insulation kinetics, as well as lower calculation costs by eliminating the need to solve the quadratic integral function [125]. For thermal debinding of ceramics via VPP, future studies should couple the pyrolysis kinetics of cured resin with heat/mass transfer processes. Additionally, the secondary reaction of uncured resin and residual stress during curing should be visible to establish more predictive models.

There are considerable experimental studies on the thermal debinding process of Si_3_N_4_ ceramics. The quality of binder removal is influenced by the chemical formulation of the photopolymer resin, the physical and morphological properties of ceramic particles, and the thermal profile parameters (e.g., heating rate and atmosphere) applied during pyrolysis [126,127].

**Figure 11:**
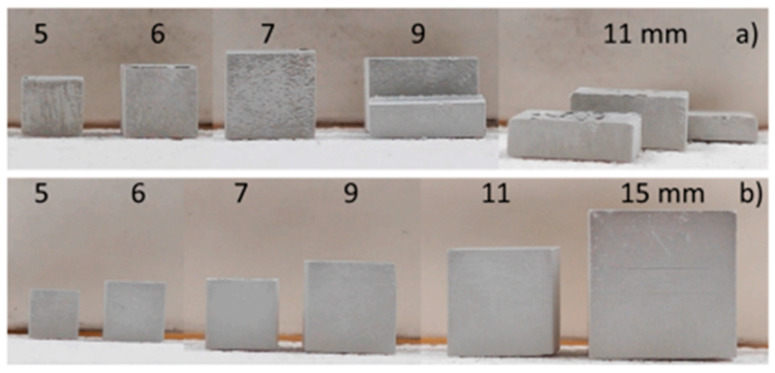
Lines of cubes that have undergone a debinding process: (**a**) profile based on the TGA at a heating rate of 10 °C/min; (**b**) profile based on the TGA at a heating rate of 0.2 °C/min, reprinted from reference [129], copyright 2025, with permission from Elsevier.

Using an atmosphere in which the process occurs through an endothermic process will always be preferable over one where the process occurs through an exothermic process [128]. It has been demonstrated by Huang et al. [73] that a bimodal particle distribution of Si_3_N_4_ enhances the green density during debinding, which increases from 1.28 g/cm^3^ to 1.37 g/cm^3^ through optimized fine/coarse particle packing. Marie et al. [129] pushed thickness boundaries by developing graded thermal programs, and achieved defect-free Si_3_N_4_ components with a 9 mm sintered wall thickness through liquid-phase crack healing—a 125% improvement over conventional limits. Figure 11 shows the lines of cubes after debinding with different heating rates [129]. Shen et al. [130] found that thermal debinding in a 95%N_2_/5%H_2_ atmosphere realizes 5 mm crack-free green bodies by moderating pyrolysis exotherms. The hydrogen fraction facilitates the hydrocarbon formation, which distributes gas evolution pressures more evenly while nitrogen stabilizes the thermal environment. This approach increased the density of sintered bodies by 18% and the flexural strength by 32% compared to air debinding.

The presence of residual carbon from incomplete binder removal has proven to be particularly detrimental. Jin et al. [131] quantified its impact, and found that carbon residues reduce the flexural strength of Si_3_N_4_ ceramics from 469 MPa to 184 MPa through the carbothermal reaction during sintering. However, an oxidation treatment at 450 °C for 5 h effectively eliminates carbon without adversely affecting the dimensional stability, which will restore the mechanical performance of Si_3_N_4_ ceramics to a comparable level.

Wang et al. [132] found that TG-FTIR-optimized protocols introduce staged heating profiles that reduced cracking in thick-walled components by 40% versus conventional methods. This study emphasized the early-stage monomer evaporation control, where rapid volatilization below 300 °C accounts for 65% of crack initiation risks [133,134].

When the debinding atmosphere is N_2_ or 95%N_2_/5%H_2_, the cracks and defects of the green body can be more effectively controlled than in air. Compared with N_2_, 95%N_2_/5%H_2_ can control the defects of the green body at a higher heating rate, which reduces the time and energy consumption of debinding, but it leads to more complicated experimental operations to control the atmosphere. In addition, the researchers suggest that the complete removal of carbon compounds can be achieved through debinding in air. Therefore, the two-step degreasing method is commonly used for VPP-based Si_3_N_4_: the organic matter is decomposed in N_2_, and then the residual carbon is discharged in air. However, the heating rate generally remains below 1 °C/min, which results in prolonged processing times and more energy expenditure.

These above-mentioned developments address the historical trade-off between geometric complexity and structural reliability in the additive manufacturing of Si_3_N_4_ ceramics. Modern debinding strategies are now feasible to produce 8–12 mm wall thicknesses with a dimensional deviation of less than 2% and achieve a relative density of more than 95%, which will be critical milestones for the industrial adoption of photopolymerization-based ceramic additive manufacturing.

### 4.2. Sintering Process

Silicon nitride primarily exists in two crystalline forms: equiaxed *α*-Si_3_N_4_ (the low-temperature stable phase) and elongated rod-like *β*-Si_3_N_4_ (the high-temperature stable phase). When heated to approximately 1420 °C, *α*-Si_3_N_4_ undergoes a reconstructive phase transformation to *β*-Si_3_N_4_. High-temperature sintering plays a pivotal role in determining the microstructure and performance of additively manufactured ceramics [135]. Table 3 summarizes the density and mechanical properties of Si_3_N_4_ ceramics produced by different sintering methods. Huang et al. [73] found that pressureless sintering at 1800 °C under nitrogen achieves optimal densification for Si_3_N_4_ components, with finer particles enhancing dimensional shrinkage through improved particle rearrangement. The main crystalline phase was *β*-Si_3_N_4_, indicating that the phase transition from the *α*- to *β-*phase was completed. Si_3_N_4_ ceramics produced by using a coarse-to-fine particle ratio of 3:7 for raw powders have a relative density of 98% through liquid-phase sintering mechanisms, and yield excellent mechanical properties such as a flexural strength of 728.7 MPa and a hardness of 14.68 GPa. The aspect ratio of grains was approximately 2.68, which was higher than other samples and induced a higher bending strength.

Meanwhile, Chen’s work [141] on DLP-printed Si_3_N_4_-SiO_2_ composites revealed exceptional microwave transparency (dielectric constant *ε* < 4 and dielectric loss tan*δ* < 0.003) when sintered at 1350 °C. The optimized composition with 61.3% SiO_2_ in Si_3_N_4_-SiO_2_ composites when sintered at 1350 °C exhibited a flexural strength of 77 MPa and a controlled dielectric stability across 8.2–18.0 GHz frequencies, which demonstrates the dual functionality for structural/functional integrated radome applications.

Dong et al. [142] tracked the variations in the properties of Si_3_N_4_-SiO_2_ ceramics in a sintering temperature range of 1250 to 1400 °C. The measured open porosity decreases from 37.5% to 4.3%, while strength increased 4-fold from 19.4 MPa to 76 MPa, with dielectric constants oscillating between 3.45 and 4.0 due to the competing crystallization and phase transformation effects. Liu et al. [136] combined KH560 surface modification with Darvan dispersants to prepare 45 vol.% Si_3_N_4_ ceramic slurries for stereolithography. A pressure-assisted sintering at 1750 °C under 5 MPa of nitrogen was then used to achieve a density of 3.28 g/cm^3^ corresponding to a relative density of 95%, matching the hardness of 14.63 GPa and fracture toughness of 5.82 MPa·m^1^/^2^ of conventionally sintered ceramics. The main crystal phase of Si_3_N_4_ ceramics was *β*-Si_3_N_4_. The applied pressure also reduces the pore sizes by 60% compared to pressureless sintering conditions, which highlights the effectiveness of hybrid sintering strategies.

Spark plasma sintering (SPS) has emerged as a rapid, energy-efficient method for producing high-performance ceramics, offering precise control over microstructural evolution [143]. Tian et al. [138] combined SPS with DLP-printed porous Si_3_N_4_, and found that increasing temperatures from 1600 °C to 1700 °C enhances the phase transformation of *α* to *β* from 19.19% to 76.72%, concurrently improving flexural strength from 109 MPa to 250 MPa. However, extended dwell times (>10 min) at peak temperatures reduce the strength by 50% due to pore coarsening, which highlights the critical balance between sintering parameters and final properties.

Wang et al. [139] leveraged pressureless sintering to achieve the full phase transformation of *α* to *β* Si_3_N_4_ at 1750 °C, and produced ceramics with a flexural strength of 613 MPa and a fracture toughness of 7.5 MPa·m^1/2^. The anisotropic grain growth observed in these components—evidenced by 15% higher *Z*-axis shrinkage—directly correlated with elongated *β*-Si_3_N_4_ grains aligning along the build direction, showcasing a microstructure–property relationship that is unique to additive manufacturing. Chen et al. [144] produced porous Si_3_N_4_ ceramics by integrating DLP with pressureless sintering at a sintering temperature of 1800 °C, which exhibited a maximum flexural strength of 367 ± 75 MPa as well as the dielectric constant of *ε* = 7.37. A high-temperature phase Y_2_Si_3_O_3_N_4_ was formed and the presence of glass phases at grain boundaries reduced porosity. The incorporation of an octet-truss architecture successfully fabricated porous ceramics with complex microstructures, and resulted in a remarkable reduction in the dielectric constant. As the measured porosity increases from 25% to 36%, flexural strength decreases from 98 ± 7 MPa to 62 ± 13 MPa, with a corresponding decline in dielectric constant values from 5.56 to 4.42.

Porous silicon nitride structures exhibit outstanding mechanical properties, excellent oxidation resistance, and remarkable thermochemical corrosion resistance, making them suitable for harsh environments. The incorporation of SiC nanowires and free carbon into porous Si_3_N_4_ via the Polymer Infiltration and Pyrolysis (PIP) method is an effective strategy for fabricating high-performance electromagnetic shielding materials [145]. Wang et al. [140] achieved a pore reduction of 97% over eight infiltration cycles via a PIP-DLP hybrid approach, yielding Si_3_N_4_ components with a density of 2.64 g/cm^3^ and a flexural strength of 162 MPa. This iterative densification strategy proves to be particularly effective for thin-walled structures of less than 2 mm, where conventional sintering struggles to eliminate internal voids.

These above-mentioned advancements demonstrate how modern sintering techniques enable precise control over ceramic microstructures and properties when coupled with additive manufacturing. Current capabilities now span across dense Si_3_N_4_ ceramics with high strengths of more than 600 MPa to functionally graded porous ceramic systems, and meet diverse industrial demands from those of thermal management to those of radar-transparent components.

### 4.3. Influence of Sintering Additives

Due to the high bond energy of Si-N bonds and the low atomic self-diffusion coefficient in Si_3_N_4_, both volume diffusion and grain boundary diffusion rates are extremely limited, making it challenging to achieve the densification of Si_3_N_4_ ceramics through conventional solid-state sintering. Therefore, liquid-phase sintering is typically employed for the fabrication of Si_3_N_4_ ceramics [146]. During liquid-phase sintering, the sintering aids react with SiO_2_ on the surface of Si_3_N_4_ powder at high temperatures to generate a liquid phase, which significantly enhances mass transfer rates and high-temperature reactions, thereby improving densification. Table 4 summarizes the relative density and mechanical properties of Si_3_N_4_ ceramics incorporated with different sintering additives.

Li et al. [147] demonstrated that the addition of MgO-Y_2_O_3_ sintering aids enhances densification by forming eutectic phases with a low melting temperature, and achieves a relative density of 99.4% and an exceptional thermal conductivity of 64.4 W·m^−1^·K^−1^ at 8 wt.% sintering aids. The synergy high cationic field strength of Y^3+^ and the fluxing action of MgO produces Si_3_N_4_ ceramic components with a flexural strength of 879 MPa and a hardness of 15 GPa, which paves the way for high-performance thermal management devices in electronics [148,149].

Si_3_N_4_ ceramics with the sintering aids of Al_2_O_3_-Y_2_O_3_-AlN in a ratio of 95:2.5:2.5 yield an optimal microstructure during LPS at 1800 °C as reported in Qin’s study [150]. The elongated *β*-Si_3_N_4_ grains in this system contribute to a flexural strength of 540 MPa and a fracture toughness of 4.92 MPa·m^1/2^ through crack deflection without degrading dimensional stability in DLP-printed geometries.

**Table 4:** The relative density and mechanical properties of Si_3_N_4_ ceramics with different sintering additives.

Ceramic Powder	Solid Loading (vol.%)	Relative Density (%)	Flexural Strength (MPa)	Hardness (GPa)	Fracture Toughness (MPa·m^1/2^)	Linear Shrinkage Range (%)	Ref.
92Si_3_N_4_ + 2.47MgO + 5.53Y_2_O_3_	-	99.4	879 ± 37	15 ± 0.4	-	-	[147]
95Si_3_N_4_ + 2.5Y_2_O_3_ + 2.5Al_2_O_3_	45	84.2 ± 10.0	540.63 ± 10.05	12.88 ± 0.52	4.92 ± 0.07	-	[150]
Si_3_N_4_ + CeO_2_	-	95.8	-	HV_10/10_ 1347.9 ± 2.4	6.57 ± 0.07	-	[151]
90Si_3_N_4_ + 3La_2_O_3_ + 7MgO	45	95.94	577 ± 16.28	-	5.84 ± 0.17	21.75–25.45	[152]
90Si_3_N_4_ + 6Y_2_O_3_ + 4Al_2_O_3_	60	98.24 ± 0.36	865.87 ± 54.35	16.70 ± 0.34	-	15.38–18.61	[90]

SLA-fabricated Si_3_N_4_ ceramics with CeO_2_ as the sintering additives followed by a field-assisted sintering approach were reported by Rao et al. [151], which achieved full densification through grain boundary sliding, where Ce segregation at interfaces was considered to promote the formation of anisotropic needle-like grains. This method eliminated intergranular phases, which resulted in SLA-printed Si_3_N_4_ ceramics with a 12% higher hardness than conventional LPS counterparts.

The sintering additive of an La_2_O_3_-MgO system reported by Zhou et al. [152] highlighted the delicate balance between additive ratios and performance. Optimized mechanical properties such as a flexural strength of 577 MPa and a fracture toughness of 5.84 MPa·m^1/2^ were achieved at a La_2_O_3_-MgO ratio of 3:7 by tailoring liquid phase viscosity and *β*-grain growth kinetics. A critical threshold of sintering additives was also established to be 9:1 for La_2_O_3_-MgO ratios, and excessive grain coarsening degraded strength by 18–22%.

According to the studies above, controlling the type and proportion of the sintering additive, as well as the sintering process parameters, enables a relative density of more than 99% with controlled grain morphologies and improves the performance of VPP-printed Si_3_N_4_ ceramic structures. The integration of computational phase diagram modeling promises further refinements in sintering protocols.

## 5. Typical Components and Structures of Photopolymerization-Based Si_3_N_4_ Ceramics

**Figure 12:**
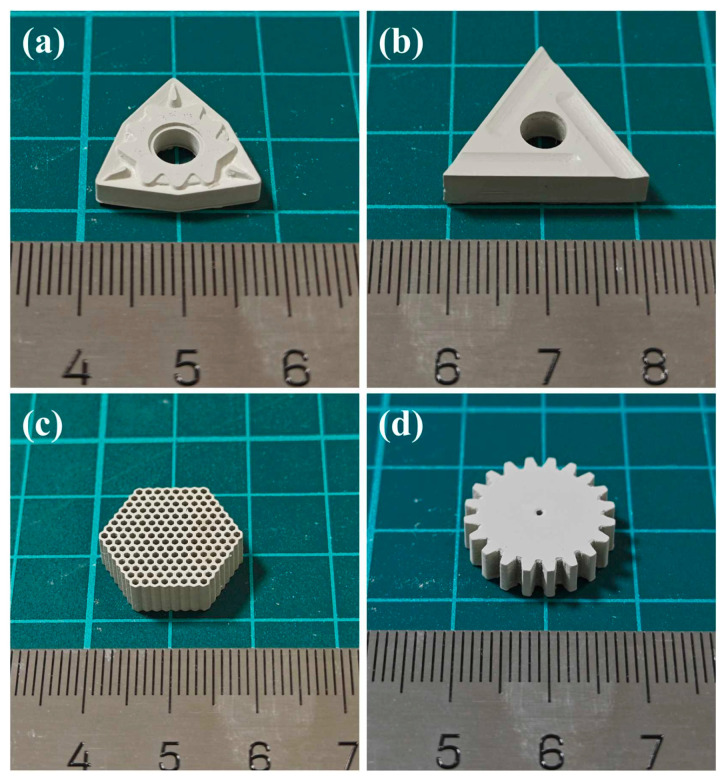
Si_3_N_4_ ceramic green bodies fabricated by vat photopolymerization: (**a**,**b**) cutting tools with chip breaker, (**c**) honeycomb structure, and (**d**) gear component, reprinted from reference [91], copyright 2025, with permission from Elsevier.

Silicon nitride (Si_3_N_4_) is a highly advantageous material due to its unique combination of mechanical, thermal, chemical, electrical, and magnetic properties under both ambient and high-temperature conditions. These characteristics enable broad applications of Si_3_N_4_ components, including in heat exchangers [153], environmental barrier coatings, osseointegration scaffolds [154], radomes, radar-absorbing materials [155], and integrated circuits. Such applications often require geometric complexity to achieve efficient and/or effective operations [144]. However, traditional ceramic processing methods, such as hot pressing or die pressing, are typically limited to simple axisymmetric shapes. The advent of additive manufacturing (AM) has led to significant advancements in fabricating geometrically complex Si_3_N_4_ components. Li et al. [91] successfully printed Si_3_N_4_ ceramic cutting tools, honeycomb structures, and gear components via vat photopolymerization, as shown in Figure 12 [91].

**Figure 13:**
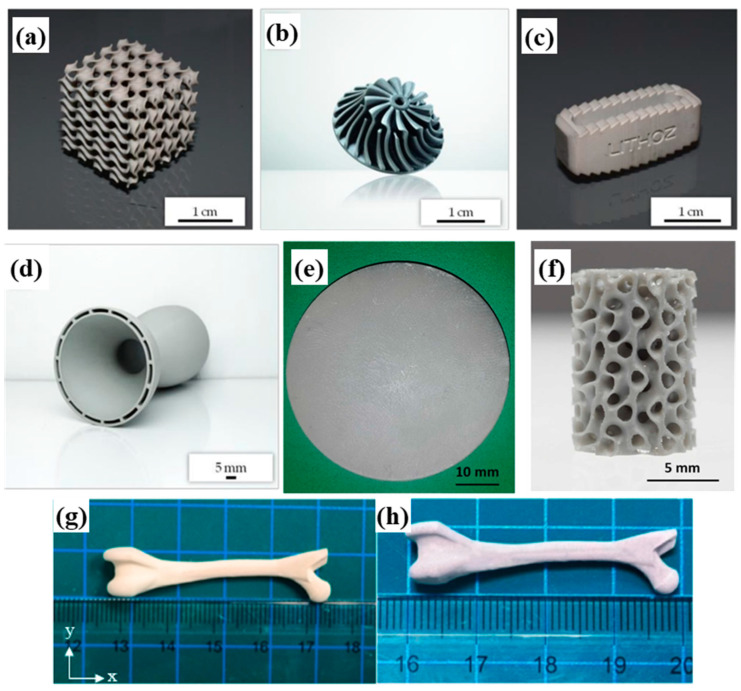
Printed Si_3_N_4_ ceramic via VPP: (**a**) gyroids; (**b**) impeller; (**c**) spinal implant; (**d**) de laval nozzle, adapted from reference [156], copyright 2020, with permission from MDPI; (**e**) disk; (**f**) TPMS SplitP component, adapted from reference [137], copyright 2023, with permission from Elsevier; (**g**,**h**) biomimetic human bone structures, adapted from reference [160], copyright 2023, with permission from Elsevier.

Altun et al. [156] revolutionized the fabrication of Si_3_N_4_ components through lithography-based ceramic manufacturing (LCM), producing intricate components like bone-mimetic scaffolds and micro-turbines with mechanical properties rivaling conventionally processed ceramics, as shown in Figure 13a–d. This breakthrough enabled the production of geometries that were unattainable via milling or molding, such as porous lattice structures optimized for bone ingrowth.

Schwarzer-Fischer et al. [137] promoted this capability by developing a photosensitive resin that is compatible with Lithoz’s CerAM VPP system. Their work successfully printed triply periodic minimal surface (TPMS) architectures for finger implants, demonstrating how controlled porosity gradients of 30 vol.% to 60 vol.% balance structural integrity such as a compressive strength of more than 50 MPa with biological functionality, as shown in Figure 13e,f.

Although silicon nitride ceramics have been widely fabricated using various 3D printing technologies over the past decade, the most common 3D-printed Si_3_N_4_ ceramic implants for biomedical applications are primarily produced via vat photopolymerization (VPP) 3D printing and material extrusion (MEX) 3D printing. Huang et al. [157] achieved a milestone in printing dense yet porous Si_3_N_4_ structures by optimizing the slurries with a high viscosity of higher than 10 Pa·s at a shear rate of 30 s^−1^. The resulting square honeycombs, with wall thickness ratios of up to 12:1, exhibited compressive strengths exceeding 120 MPa at a relative density of 40%, which was considered to outperform traditional ceramic foams through crack-deflection mechanisms enabled by engineered surface roughness.

In the biomedical field, silicon nitride exhibits stable and excellent biocompatibility, with remarkable advantages in antibacterial performance, which achieves an antibacterial rate of 94.6%. Furthermore, the surface of Si_3_N_4_ parts supports a well-defined cell morphology and normal migration, which significantly enhances cell spreading, adhesion, and intercellular crosslinking [158]. Silicon nitride is also an emerging implant material with promising potentials for human hard tissue replacements [159]. Huang et al. [160] fabricated biomimetic human bone structures of Si_3_N_4_ via DLP, as shown in Figure 13g,h. DLP-printed parts developed by Zou et al. [161] highlighted the clinical potential of silicon nitride for dental implant applications. These devices have a flexural strength of 770 MPa and a fracture toughness of 13.3 MPa·m^1/2^, which are comparable with machined Si_3_N_4_ ceramics; in the meantime, they also demonstrate an antibacterial efficacy of 94.6% against oral pathogens. Cytocompatibility tests revealed normal fibroblast proliferation, with no hemolytic reactions or mucosal irritation, which fulfills the ISO 10993 standard for implantable devices.

Feng et al. [162] refined the manufacturing pipeline by developing Si_3_N_4_ ceramic slurries with a low viscosity of 1.95 Pa·s at 30 s^−1^ at a solid loading of 40 vol.%. Pressure-assisted sintering at a temperature of 1800 °C and an applied pressure of 0.4 MPa in a N_2_ atmosphere yielded implants with a relative density of 96.08% and a fracture toughness of 5.88 MPa·m^1/2^. Six-week in vivo trials of Si_3_N_4_ implants showed successful osseointegration, with histological analysis confirming <5% fibrous tissue formation at bone–implant interfaces.

These innovations position Si_3_N_4_ as a next-generation biomaterial, with ongoing research focused on scaling production while maintaining the precision required for FDA-approved implants. The integration of AI-driven topology optimization promises to accelerate the design of patient-specific devices that harmonize mechanical, biological, and imaging requirements.

## 6. Future Opportunities and Challenges

Vat photopolymerization 3D printing of silicon nitride holds significant promise across industries requiring high-performance structural/functional ceramic components. Its exceptional thermal stability, wear resistance, and biocompatibility make it ideal for aerospace components like turbine blades, medical implants such as spinal fusion devices, and advanced electronics for heat-dissipating substrates. The technology also enables intricate water treatment modules (e.g., filtration membranes) and lightweight, durable robotics parts like wear-resistant gears. Additionally, the growing interests in multi-materials printing opens doors for hybrid structures combining Si_3_N_4_ with metals or polymers or fibers for tailored mechanical and functional properties. Four-dimensional printing has also emerged as a research hotspot. The development of ceramic 4D printing has progressed from elastomeric homogeneous precursors toward heterogeneous precursors based on single- or multi-material printing [163]. It is expected that 4D printing can be more widely applied to Si_3_N_4_ ceramics in the future, thereby achieving enhanced mechanical properties. Here are the following challenges for Si_3_N_4_ based on vat photopolymerization:

(1) Creating stable and high-quality Si_3_N_4_ ceramic slurries requires balancing multiple factors. Achieving an optimal slurry solid loading of typically 50–60% ceramic content is critical—a low solid concentration causes weak interlayer bonding and defects like cracks, while excessive solid loading leads to poor resin flow, particle clumping, and uneven curing [164,165]. Photopolymer resins must also chemically “wrap around” ceramic particles without separating, demanding precise dispersants and viscosity modifiers. Limited UV penetration in concentrated Si_3_N_4_ slurries further complicates uniform curing, risking incomplete layers or structural weaknesses.
(2) Every step of the printing process demands tight calibration. Exposure time, layer thickness, and light intensity must align perfectly to avoid over-curing (brittle parts) or under-curing (collapsed features). Support structures for complex geometries often leave surface marks or require tedious post-removal refinishing. Additionally, slight variations in ambient temperature or resin aging can alter curing behavior, demanding constant monitoring. For multi-material designs, mismatched thermal expansion between Si_3_N_4_ and other materials risks delamination during subsequent heat treatments.
(3) Removing organic binders through various debinding techniques requires ultra-slow heating to prevent cracks from trapped gases, while sintering must balance high temperatures of 1700–1800 °C with nitrogen atmospheres to avoid the decomposition of silicon nitride. Even minor deviations in these steps can introduce voids, warping, or weakened grain boundaries. Secondary processes like polymer infiltration (PIP) increase additional costs and complexity but remain quite necessary to achieve full densification in critical applications.
(4) In high-precision industries like aerospace and those of biomedical implants, vat photopolymerization-based Si_3_N_4_ ceramics face challenges in controlling dimensional shrinkage and surface roughness due to inherent material behavior during manufacturing. Anisotropic shrinkage from photopolymerization and sintering, combined with phase transformations, causes structural inaccuracies in critical components. Addressing these issues requires optimizing materials and post-processing techniques to align Si_3_N_4_ ceramics with the precision demands of advanced technologies.

Despite these hurdles, advancements in resin chemistry (e.g., low-organic formulations) and hybrid techniques like combining vat photopolymerization with binder jetting or robocasting offer paths forward. By minimizing carbon residues from traditional resins, these formulations prevent harmful byproducts during sintering while improving dimensional stability. Hybrid binder systems allow for the precise control of sintering aids and complex geometries, combining the high resolution of light-based printing with the structural robustness of particle-dense pastes. Such systems address shrinkage and roughness by engineering multi-scale porosity and enhancing interfacial bonding. Researchers are also exploring AI-driven process optimization to mitigate human error in parameter tuning. As the technology matures, the 3D printing of Si_3_N_4_ ceramics could revolutionize industries where precision, strength, and thermal resilience are non-negotiable—from next-generation jet engines to biodegradable bone scaffolds.

## 7. Conclusions

This paper introduces the mechanisms of vat photopolymerization and summarizes the strategies for improving Si_3_N_4_ ceramic slurries as well as controlling the printing and debinding/sintering processes. It further highlights the ways in which different approaches can be used to enhance the properties of Si_3_N_4_ slurries and ceramic parts. Finally, applications of vat photopolymerization-based Si_3_N_4_ ceramics and composites in various fields such as those of aviation, aerospace, energy, electronics, chemical processes, and biomedical implants are also presented to point out future opportunities and challenges. The main conclusions are as follows:

(1) The mechanisms of vat photopolymerization rely on UV light to selectively cure photopolymer resins mixed with ceramic powders. However, due to the refractive index difference between ceramics and resins, light scattering limits curing depth and resolution at high solid loadings, which reduces print fidelity. In addition, polymerization shrinkage induces internal stresses, which causes delamination or microcracks in green bodies during debinding and sintering.
(2) This review highlights the interplay between material properties, printing parameters, and post-processing protocols. Key advancements include optimizing ceramic slurries through particle size gradation, refractive index matching, and surface modifications to mitigate challenges posed by the high refractive index of Si_3_N_4_ and UV absorption. Strategies such as bimodal particle distributions and high-refractive-index resins enhance the slurry stability, curing depth, and mechanical performance.
(3) The integration of composite reinforcements, such as whiskers and fibers, can enhance the mechanical properties of VPP-based Si_3_N_4_ ceramics. PDCs also offer a unique pathway to ceramic fabrication via 3D printing techniques such as VPP, bypassing the need for sintering aids.
(4) Debinding and sintering processes are tailored to minimize defects, with innovations like nitrogen-hydrogen atmospheres and pressure-assisted sintering achieving a relative density of higher than 95%. The thermal debinding process of ceramics via VPP can be optimized by establishing predictive models. Future studies should couple the pyrolysis kinetics of cured resin with heat/mass transfer processes. Controlling the type and proportion of the sintering additive can improve the performance of VPP-printed Si_3_N_4_ ceramic structures.

In conclusion, VPP-based 3D printing has unlocked new frontiers in Si_3_N_4_ ceramic manufacturing. Continued progress in material science, process engineering, and AI-driven computational modeling will further bridge the gap between laboratory research and industrial-scale production, which enables the creation of next-generation high-performance Si_3_N_4_ ceramic components.

## Figures and Tables

**Table 2:** Viscosity and curing properties of Si_3_N_4_ slurries with different surface modifications.

Surface Modification Methods		D_50 _ (μm)	Solid Loading (vol.%)	Viscosity (Pa·s)	Curing Depth (μm)	S_d_ (μm)	E_d_ (mJ/cm^2^)	Ref.
Surface oxidation	800 °C 24 h	0.5	25	0.068 at 30 s^−1^	52	-	-	[42]
	1200 °C 1 h	0.6	50	7.268 at 100 s^−1^	80	-	-	[87]
	1200 °C 1.5 h	0.7	35	-	100	-	-	[89]
	1200 °C 3 h	0.2	-	-	68	-	-	[88]
Surface modifier	KH560	0.83	40		45			[72]
	KMT-3331(2 wt.%)	0.7	50	3.1 at 30 s^−1^	-	8.39	-	[90]
Surface coating	YAG via NCP ^a^	0.7	30	-	-	9.42	3.28	[91]
	Bowl-like boehmite(6 wt.%)	1.25	40	<2 at 30 s^−1^	40	21.4	4.85	[92]
	Thermosetting resin E51(5 wt.%)	0.83	40	2 at 30 s^−1^	61.6	-	-	[86]

^a^ NCP: Non-aqueous chemical precipitation.

**Table 3:** The densities and mechanical properties of Si_3_N_4_ ceramics produced by different sintering method.

Sintering Methods		D_50 _ (μm)	Solid Loading (vol.%)	Density	Flexural Strength (MPa)	Hardness (GPa)	Fracture Toughness (MPa·m^1/2^)	Ref.
Dry-pressed sintering	1750 °C 5 MPa 2 h 10 °C/min	0.8	45	95%	-	14.63 ± 0.45	5.82 ± 0.42	[136]
	1775 °C 5 MPa 2 h	0.5	40	99 ± 0.05%	847	-	-	[137]
SPS	N_2_ 1700 °C 5 min 100 °C/min	1.19	-	-	249.5 ± 4.0 (Porous structure)	-	-	[138]
Pressureless sintering	N_2_ 1750 °C 2 h 5 °C/min	0.84	48	3.09 g/cm^3^	613.3 ± 53.1	12.6 ± 0.4	7.5 ± 0.3	[139]
	N_2_ 1800 °C 2 h	0.7	43	98.88%	833.74	15.75 ± 0.20	5.17 ± 0.25	[91]
	1825 °C 4 h	0.7	40	3.21 g/cm^3^	701.66	13.81	5.34	[94]
PIP	8 infiltration cycles	0.84	55	2.64 g/cm^3^	162.35	-	-	[140]

## Data Availability

No new data were created or analyzed in this study.
